# The Impact of COVID-19 on Psychiatric Emergency and Inpatient Services in the First Month of the Pandemic in a Large Urban Mental Health Hospital in Ontario, Canada

**DOI:** 10.3389/fpsyt.2021.563906

**Published:** 2021-04-23

**Authors:** Helena K. Kim, Andre F. Carvalho, David Gratzer, Albert H. C. Wong, Shayla Gutzin, M. Ishrat Husain, Benoit H. Mulsant, Vicky Stergiopoulos, Zafiris J. Daskalakis

**Affiliations:** ^1^Department of Psychiatry, University of Toronto, Toronto, ON, Canada; ^2^Department of Psychiatry, Centre for Addiction and Mental Health, University of Toronto, Toronto, ON, Canada

**Keywords:** COVID-19, psychiatry, Canada, pandemic, emergency, inpatient, hospital

## Abstract

The World Health Organization characterized COVID-19 (coronavirus disease 2019) as a pandemic on March 11, 2020 (WHO). Within a couple of days, all Canadian provinces announced the implementation of social distancing measures. We evaluated the immediate effect of COVID-19 on psychiatric emergency and inpatient services in Canada's largest psychiatric hospital in the first month of the pandemic. We extracted data from the electronic medical records of the Center for Addiction and Mental Health in Toronto, Canada. We compared emergency department visits, inpatient occupancy rates, and length of stay in March 2019 and March 2020, and during the first and second half of March 2020. There was a decrease in the number of emergency department visits and inpatient occupancy rates in March 2020 compared to March 2019. There was also a significant decrease in the number of emergency department visits and inpatient occupancy rates in the second half of March 2020 compared to the first half. Our findings suggest that the pandemic was followed by a rapid decrease in the usage of psychiatric emergency and inpatient services in a large mental health hospital. Future studies will need to assess whether this decrease will be followed by a return to baseline or an increase in need for these services.

## Introduction

COVID-19 (Coronavirus disease 2019) is a disease caused by the SARS-CoV2 (1) virus identified in Wuhan, China in December 2019. It was declared a pandemic on March 11th. Within 2 months, over 316,000 deaths have been confirmed globally (WHO). Several recent papers have hypothesized that patients with psychiatric and/or substance use disorders may be particularly vulnerable to being infected with COVID-19 and to experience adverse outcomes, emphasizing the importance of studying the effect of this pandemic in this vulnerable population (2–6).

Several studies have examined the effect of disasters on mental health. After natural disasters, psychotropic prescription fills or supply decreased (7–9). By contrast, terrorist attacks increased mental health service use (10–12). Similarly, some studies have reported an increase in the use of psychiatric services among the survivors of the 2003 severe acute respiratory syndrome (SARS) pandemic (13, 14). However, during the SARS pandemic itself, there was a significant shift from hospitals to community clinics for anxiety disorders that was attributed to the perception that the risk of being infected if one was hospitalized outweighed the potential benefit of a psychiatric hospitalization (15). A recent review examining the effects of quarantine in light of self-isolation of individuals who are potentially exposed to COVID-19 noted negative psychological consequences of quarantine, including post-traumatic stress symptoms, some of which can be long-lasting (16). It has also been speculated that social distancing associated with the current COVID-19 pandemic may both directly and indirectly increase the risk of suicide (17). Together, these studies show that the potential impact of disasters on the need for, and use of, psychiatric services is complex.

On March 16, 2020, provinces of Canada declared the COVID-19 pandemic a state of emergency, ordering closures of non-essential services and prohibiting large public gatherings. The Center for Addiction and Mental Health (CAMH) in Toronto, Ontario is the largest mental health hospital in Canada. It provides care to more than 34,000 patients each year and is in the metropolitan Toronto, which is the most populous metropolitan area in Canada (statscan.gc.ca). After March 16, we noticed a decrease in the number of both visits in the psychiatric emergency department (ED) and admissions at CAMH, suggesting that COVID-19 was possibly affecting the way in which patients access and use psychiatric services. To test this hypothesis, we examined the number of ED visits and the occupancy rates on acute inpatient units in the month of March 2020. To our knowledge, this paper is the first consideration of the impact of COVID on mental health utilization in a psychiatric hospital in Canada. While we only examined one hospital, due to the size of CAMH and the acuity of the COVID-19 outbreak in metropolitan Toronto (https://www.toronto.ca/home/covid-19), we hope that the findings of this study will contribute to increasing our currently limited body of knowledge on how pandemics affect psychiatric care utilization in hospitals in urban settings.

## Materials and Methods

In this natural study, we used a pre-post study design (18) to test the hypothesis that the usage of both ED and inpatient psychiatric services in March 2020 was lower than in March 2019 and that the decrease would be attributable to a decrease from the first to the second half of March 2020. We chose March because the COVID-19 pandemic was declared an emergency on March 16 in Canada. In comparing 2 weeks prior to and after the closure of non-essential services, we hoped to identify the immediate effect of the pandemic in psychiatric service utilization within the same month. In doing so, we hoped to compare two short time frames that would have similar weather conditions and other sociopolitical factors influencing service utilization outside of acute changes produced by the pandemic.

### Study Sites

Data from this study were extracted from electronic medical records (EMR) of the CAMH ED and its 10 acute inpatient units: Acute Care Unit A (ACU A), Concurrent Addictions Inpatient Treatment Service (CAITS), Emergency Assessment Unit (EAU), Early Psychosis Unit (EPU), General Psychiatric Unit (GPU) A and B, Mood and Anxiety Inpatient Unit (MAUI), Medical Withdrawal Services (MWS), Psychiatric Intensive Care Unit (PICU), and Women's Inpatient Unit (WIU). CAITS and MWS are inpatient units for concurrent addiction services. ACU and PICU are inpatient units for patients of higher acuity.

### Data Collection

For March 2020, we extracted the number of daily ED visits for March and the daily bed occupancy rates for March for all 10 inpatient units combined and for each of the individual inpatient units listed above. We also extracted: the median time between registering in the CAMH ED and completing triage (triage), the median time between registering in the ED and either be admitted for those who were admitted (arrival to admission) or leaving the ED (arrival to leave ED), the median length of stay (LOS) on inpatient units, and the number of inpatient discharges from all inpatient units. We compared these variables during two different time periods: from March 1 to 15 and from March 16 to 31. These two periods were chosen as March 16 was when quarantine and social distancing measures were first announced in Canada.

For March 2019, we extracted the total number of ED visits, the acute inpatient monthly occupancy rate, which is the combined inpatient occupancy rate of all 10 acute inpatient units mentioned above, and the monthly occupancy rate for each of the individual inpatient units.

### Statistical Analysis

Statistical analysis was performed to compare the daily number of ED visits, daily occupancy rates for all acute inpatient units combined, and daily occupancy rates for each individual inpatient unit between March 1–15, 2020 and March 16–31, 2020. Data from each day was treated as an independent observation. Kolmogorov-Smirnov test was used to examine if the data was normally distributed. Mann-Whitney (MW) U test was used to compare the two time periods. Data is presented as median ± interquartile range (IQR). IBM SPSS Statistics 26 was used for statistical analysis.

### Descriptive Comparisons

Monthly occupancy rate for all acute inpatient units combined was calculated by dividing the total number of days spent by all the patients on all inpatient units during March by the number of days in March (31) and the total number of beds in all acute inpatient units (148). Monthly occupancy rates for the individual inpatient units were calculated similarly using the number of days spent on each inpatient unit and the number of beds on each unit.

We calculated the percent change between March 2019 and March 2020 for total number of ED visits and inpatient monthly occupancy rates.

The extracted data for triage duration, arrival to admission, arrival to leave ED, and inpatient LOS were median values for the following two time periods: March 1–15, 2020 and March 16–31, 2020. We did not have their interquartile ranges or daily values, allowing for a descriptive comparison between the two extracted medians but not a statistical comparison. Therefore, we calculated the percent change between March 1–15, 2020 and March 16–31, 2020 for the following: total ED visits, triage duration, ED arrival to admission, ED arrival to leave, inpatient LOS, and number of inpatient discharges.

## Results

### Number of ED Visits

#### Descriptive Comparisons

The number of ED visits decreased 27% from 1,305 visits in March 2019 to 949 in March 2020.

The descriptive comparison of the ED visit between the first and second half of March 2020 are presented in Table 1: a 25% decrease in total ED visits, 41% decrease in triage duration, 29% decrease in the time from arrival to admission, and 39% decrease in the time from arrival to leaving the ED was found.

**Table 1:** Comparison of ED visit data, length of stay (LOS) and total number of discharges in acute inpatient units between March 1–15 and March 16–31, 2020.

	**March 1th−15th**	**March 16th−31st**	**Percent change**
Number of total ED visits	545	404	−25%
Triage (hours)	1.7	1.0	−41%
Arrival to admission (hours)	9.2	6.5	−29%
Arrival to leaving the ED (hours)	4.6	2.8	−39%
LOS on inpatient units (days)	5	8	+60%
Number of inpatient discharges	176	177	0%

* *All numbers presented are medians, except for numbers of ED visits and number of inpatient discharges. Triage represent the amount of time between when patients register in the ED and when they are assessed by a psychiatrist or an allied health staff at the ED. Arrival to admission represent the time between when patients register in the ED and when they are admitted the ED for those who are admitted*.

#### Statistical Comparisons Between the First and Second Half of March, 2020

**Figure 1:**
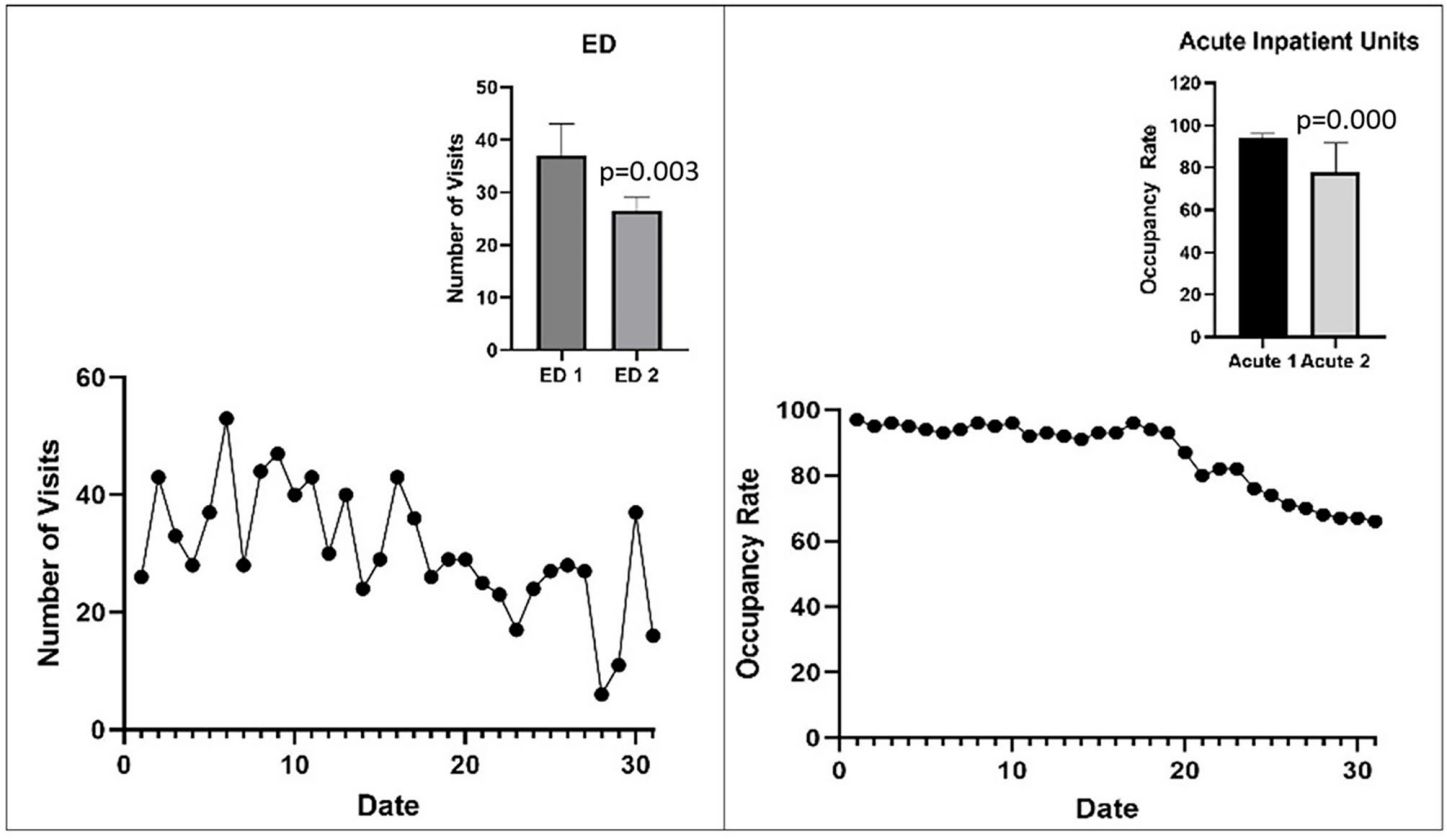
Line graphs represent the number of ED visits and the overall occupancy rates in 10 acute inpatient units each day in March, 2020. The bar graphs represent the median number of ED visits or median daily occupancy rate in the first half of March 2020 (labeled ED 1 and Acute 1, respectively) and the second half of March 2020 (labeled ED 2 and Acute 2, respectively). Error bars represent interquartile range. Statistical significance was determined with the Mann-Whitney *U* test (see text).

The median number of ED visits per day was significantly lower in the second half (27 ± 6) compared to the first half (37 ± 15, MW U = 44.5, *p* = 0.003). Figure 1 presents the line graph for daily ED visits in the month of March, and a bar graph comparing the first and second half of March for the median number of daily ED visits.

### Inpatient Occupancy Rates

#### Descriptive Comparisons

Descriptive comparisons of occupancy rates between March 2019 and 2020 are presented in Table 2. There was a 10% decrease in the combined occupancy rate of acute inpatient units. The two high acuity units (ACUA and PICU) had an increase in occupancy rate, while the other eight units (CAITS, EAU, EPU, GPU A, GPU B, MAUI, MWS, and WIU) had a decrease in occupancy rate.

**Table 2:** Monthly occupancy rates for March 2019 and March 2020.

	**Number of beds**	**Occupancy rate for March 2019**	**Occupancy rate for March 2020**	**Percent change**
Combined	148	95.8%	86.4%	−10%
ACU A	6	97.5%	98.3%	+1%
CAITS	20	94.2%	88.1%	−7%
EAU	12	81.3%	63.8%	−22%
EPU	20	95.8%	86.0%	−10%
GPU A	22	97.7%	87.5%	−10%
GPU B	20	99.1%	85.7%	−14%
MAUI	13	99.4%	95.0%	−4%
MWS	12	96.3%	86.3%	−10%
PICU	3	82.8%	98.4%	+19%
WIU	20	99.5%	87.2%	−12%

* *Monthly occupancy rate was calculated by dividing the total number of days spent by all the inpatients by the multiple of number of days in March (31) and the total number of beds (148). The retrospective nature of data collection only allowed for the extraction of a single value for occupancy rate for the entire month of March 2019. This was the same for March 2020. Because these two values could not be compared using a statistical test, we used percent change to provide a descriptive measure of change between the 2 years*.

*ACU A, acute care unit A; CAITS, concurrent addictions inpatient treatment service residential treatment unit; EAU, emergency assessment unit; EPU, early psychosis unit; GPU, general psychiatric unit; MAUI, mood and anxiety inpatient unit; MWS, medical withdrawal service; MW U, Mann-Whitney U; PICU, psychiatric intensive care unit; WIU, women's inpatient unit*.

Descriptive comparisons for inpatient LOS and number of inpatient discharges between the first and second half of March are also presented in Table 1. The median LOS increased from 5 days to 8 days, while the number of discharges did not change.

#### Statistical Comparisons Between the First and Second Half of March, 2020

Figure 1 presents the line graph for the daily occupancy rate for all inpatient units combined and the bar graph representing between-group differences for the two time periods. Table 3 presents the statistical comparison of median daily occupancy rates in the acute inpatient units. There was a significant decrease in the combined occupancy rates of all acute inpatient units. The median daily occupancy rates did not change in the ACUA, MAUI, and PICU, while it decreased significantly in the CAITS, EAU, EPU, GPU A, GPU B, MWS, and WIU (see Figures in supplementary material).

**Table 3:** Median daily occupancy rates on acute inpatient units during the first and second half of March 2020.

	**Occupancy rate in 1st half**	**Occupancy rate in 2nd half**	**MW U, *p***
Combined	94 ± 3%	80 ± 23%	MW U = 28.5, *p* = 0.000
ACU A	100 ± 0%	100 ± 7%	MW U = 84.0, *p* = 0.086
CAITS	99 ± 8%	88 ± 23%	MW U = 14.0, *p* = 0.000
EAU	76 ± 13%	38 ± 30%	MW U = 24.0, *p* = 0.000
EPU	93 ± 12%	87 ± 33%	MW U = 62.5, *p* = 0.023
GPU A	96 ± 6%	86 ± 27%	MW U = 41.5, *p* = 0.002
GPU B	94 ± 3%	73 ± 27%	MW U = 51.0, *p* = 0.006
MAUI	99 ± 8%	98 ± 15%	MW U = 103.0, *p* = 0.491
MWS	92 ± 6%	83 ± 17%	MW U = 58.0, *p* = 0.014
PICU	100 ± 0%	100 ± 3%	MW U = 103.0, *p* = 0.329
WIU	100 ± 2%	67 ± 35%	MW U = 55.5, *p* = 0.009

* *All data are median ± interquartile range*.

*ACU A, acute care unit A; CAITS, concurrent addictions inpatient treatment service residential treatment unit; EAU, emergency assessment unit; EPU, early psychosis unit; GPU, general psychiatric unit; MAUI, mood and anxiety inpatient unit; MWS, medical withdrawal service; MW U, Mann-Whitney U; PICU, psychiatric intensive care unit; WIU, women's inpatient unit*.

## Discussion

The COVID-19 pandemic is an international public health crisis (WHO) with potentially significant implications for patients with psychiatric disorders (3–6). We compared ED visits and inpatient occupancy rates between March 2019 and March 2020 and between the first and second half of March 2020 in the largest mental health hospital in Canada. Our study focused on the 2 weeks before and after the announcement of social distancing measures to allow for the examination of immediate changes produced by the implementation of these measures compared to the same time frame that would presumably have the same weather conditions and sociopolitical factors influencing service utilization. We found a significant decrease in the number of ED visits and occupancy rates overall and on all the units except for three, two of which have the highest level of acuity. These findings demonstrate the immediate impact of pandemic-related social distancing measures on emergency and inpatient psychiatric care utilization in the largest mental health hospital in Canada.

Psychiatry ED visits decreased by 27% in March 2020 compared to March 2019. There was a similar decrease (25%) between the first and second half of March 2020, suggesting an acute change in service utilization within the same month. It is important to note that our findings are limited in its generalizability as it was derived from one hospital. However, similar changes were observed in a metropolitan hospital in Portugal, showing a rapid decrease in psychiatric ED visits within two weeks of the emergency state period in Portugal (19) and within a month in a large tertiary hospital in Connecticut (20). Furthermore, a cross-sectional study of 24 EDs in five States observed a steep decline in the number of ED visits after the rise in COVID-19 cases, with the first week of mid-March being the most significant (21). Interestingly, Goncalves-Pinho and colleagues also reported that ED visits steadily increased after the first 2 weeks (19), suggesting that the impact of pandemic-related social restrictions on service utilization may be most acute in the beginning. These findings together suggest that in future waves or pandemics, clear public messaging regarding the need for patients to continue to seek psychiatric care as appropriate may be important prior to the implementation of social distancing measures. This is also congruent with the shift away from psychiatric admissions observed during the SARS pandemic (15).

The decreases in the triage time in the ED, time from arrival to admission, or time from arrival to leaving the ED reflect the lower number of patients. It is also possible that patients were assessed more quickly by the staff who wished to minimize the risk of virus transmission in the ED.

In March 2020, there was an overall decrease in the monthly occupancy rate for all acute inpatient units combined, compared to March 2019. This overall decrease reflects a decrease on all the inpatient units except for the 2 that cater to patients with the highest acuity. The same pattern was observed when comparing daily occupancy rates during the first and second half of March. These decreases in the number of occupancy rates may be due to the observed decrease in the number of ED visits or a higher threshold applied when deciding to admit a patient to minimize the risk of a COVID-19 infection.

By contrast, the total number of discharges did not differ between the first and second half of March, suggesting that decisions to discharge a patient were not affected by COVID-19. Similarly, the absence of a decrease in occupancy rates on the two units for patients of higher acuity (ACU and PICU), suggests that the admission and discharge of these patients is not affected by COVID-19. The median LOS in inpatient units was 3 days longer in the second half of March 2020 compared to the first half. This could be because patients who were admitted were more ill and require longer admissions; alternatively, it could be because fewer beds were occupied and the need to discharge patients were lower.

Also, patients at CAMH ED or EAU who are suitable for ACU or PICU are often admitted to general wards due to the small number of beds in the acute units. These patients may now be more readily admitted to the acute units due to the lower number of patients from the ED awaiting transfer to inpatient units. Furthermore, COVID-19 related changes may be interfering with discharge planning (i.e., housing, arranging follow-up social and medical care), causing psychiatrists to have a higher threshold in discharging a patient.

The findings of this study should be interpreted in light of some limitations. First, as this was a retrospective study, the data we could extract from the health records were limited and allowed only for descriptive comparisons. That is, apart from daily occupancy rates and ED visits, the majority of the extracted data were median values for a specified time frame, allowing only for descriptive comparisons. More importantly, our findings are limited in that it examines a short time frame in one hospital. This limits the generalizability of these findings to other settings. Future studies should examine longer timeframes in multiple hospitals to characterize the delayed impact of the pandemic on psychiatric care utilization across the country.

In conclusion, our findings suggest that the COVID-19 pandemic led to a rapid decrease in ED and inpatient services in a large mental health hospital. This may reflect a complex interplay among patients (e.g., a higher threshold to come to the ED and seek admission) and providers (e.g., a higher threshold to admit). Many of these pandemic-related changes in both patient and provider behavior can be interpreted as rational responses to the rebalancing of risk-benefit calculations for seeking or providing psychiatric care during a pandemic. Previous studies and some expert opinion (13, 14, 17, 22) suggest that this decrease in utilization of psychiatric services may have long-term consequences. Future studies should examine potential confounding clinical and demographic factors and a wider range of clinical settings, geographical area, and timeline, which may add important insights. Increasing our knowledge in how pandemics affect psychiatric care utilization may contribute to preparing for similar crises in the future to provide better care for this vulnerable population.

## Data Availability Statement

The raw data supporting the conclusions of this article will be made available by the authors, without undue reservation.

## Ethics Statement

Ethical review and approval was not required for the study on human participants in accordance with the local legislation and institutional requirements. Written informed consent for participation was not required for this study in accordance with the national legislation and the institutional requirements.

## Author Contributions

HK and ZD contributed to data collection, data analysis, and manuscript writing. AC, DG, AW, MH, BM, and VS contributed to data analysis and manuscript writing. SG contributed to data collection and manuscript writing. All authors approved the final version of the manuscript.

## Conflict of Interest

MH is a PI for a trial sponsored by COMPASS Pathways Limited for which he receives salary support. MH has been awarded grants from the Brain and Behavior Research Foundation, Physician's Services Incorporated Foundation, Stanley Medical Research Institute and University of Toronto. BM reports research financial support from Brain Canada, CAMH Foundation, Canadian Institutes for Health Research, and US National Institutes of Health; nonfinancial support from Pfizer (medication for an NIH-funded trial), Eli Lilly (medication and matching placebo for an NIH-funded trial), Capital Solution Design LLC (software for a trial funded by the CAMH Foundation), and HAPPYneuron (software for a trial funded by Brain Canada). In the last 5 years, ZD has received research and equipment in-kind support for an investigator-initiated study through Brainsway Inc and Magventure Inc. ZD work is supported by the Canadian Institutes of Health Research (CIHR), the National Institutes of Mental Health (NIMH), Brain Canada, and the Temerty Family and Grant Family through the Centre for Addiction and Mental Health (CAMH) Foundation and the Campbell Institute. AW's funding sources include CIHR and Miner's Lamp Award. The remaining authors declare that the research was conducted in the absence of any commercial or financial relationships that could be construed as a potential conflict of interest.

## References

[1] Andersen KG Rambaut A Lipkin WI Holmes EC Garry RF. The proximal origin of SARS-CoV-2. Nat Med. (2020) 26:450–2. 10.1038/s41591-020-0820-9 32284615 PMC7095063

[2] Farhoudian A Baldacchino A Clark N Gerra G Ekhtiari H Dom G. COVID-19 and substance use disorders: recommendations to a comprehensive healthcare response. An International Society of Addiction Medicine Practice and Policy Interest Group Position. Paper Basic Clin Neurosci. (2020) 11:133–50. 10.32598/bcn.11.covid19.1 32855772 PMC7368103

[3] Kozloff N Mulsant BH Stergiopoulos V Voineskos AN. The COVID-19 global pandemic: implications for people with schizophrenia and related disorders. Schizophrenia Bulletin. (2020) 46:752–7. 10.1093/schbul/sbaa051 32343342 PMC7197583

[4] Xue SHI Ortiz A Husain O Daskalakis ZJ Mulsant BH. COVID-19: implications for people with bipolar disorder. J Affect Disord. (2020). 10.1177/2050312120981178

[5] Yao H Chen JH Xu YF. Patients with mental health disorders in the COVID-19 epidemic. Lancet Psychiatry. (2020) 7:e21. 10.1016/S2215-0366(20)30090-0 32199510 PMC7269717

[6] Zhu Y Chen L Ji H Xi M Fang Y Li Y. The Risk and Prevention of Novel Coronavirus Pneumonia Infections Among Inpatients in Psychiatric Hospitals. Neurosci Bull. (2020) 36:299–302. 10.1007/s12264-020-00476-9 32096116 PMC7056754

[7] Fried BJ Domino ME Shadle J. Use of mental health services after hurricane Floyd in North Carolina. Psychiatr Serv. (2005) 56:1367–73. 10.1176/appi.ps.56.11.1367 16282254

[8] Quast T Gregory S Storch EA. Utilization of Mental Health Services by Children Displaced by Hurricane Katrina. Psychiatr Serv. (2018) 69:580–6. 10.1176/appi.ps.201700281 29334877 PMC11227124

[9] Williams AR Tofighi B Rotrosen J Lee JD Grossman E. Psychiatric comorbidity, red flag behaviors, and associated outcomes among office-based buprenorphine patients following Hurricane Sandy. J Urban Health. (2014) 91:366–75. 10.1007/s11524-014-9866-7 24619775 PMC3978155

[10] Boscarino JA Adams RE Figley CR. Mental health service use after the World Trade Center disaster: utilization trends and comparative effectiveness. J Nerv Ment Dis. (2011) 199:91–9. 10.1097/NMD.0b013e3182043b39 21278537 PMC3334529

[11] DiMaggio C Galea S Richardson LD. Emergency department visits for behavioral and mental health care after a terrorist attack. Ann Emerg Med. (2007) 50:327–34. 10.1016/j.annemergmed.2006.10.021 17145111

[12] Stene LE Dyb G. Health service utilization after terrorism: a longitudinal study of survivors of the 2011 Utoya attack in Norway. BMC Health Serv Res. (2015) 15:158. 10.1186/s12913-015-0811-6 25890344 PMC4457986

[13] Mak IW Chu CM Pan PC Yiu MG Chan VL. Long-term psychiatric morbidities among SARS survivors. Gen Hosp Psychiatry. (2009) 31:318–26. 10.1016/j.genhosppsych.2009.03.001 19555791 PMC7112501

[14] Tansey CM Louie M Loeb M Gold WL Muller MP de Jager J. One-year outcomes and health care utilization in survivors of severe acute respiratory syndrome. Arch Intern Med. (2007) 167:1312–20. 10.1001/archinte.167.12.1312 17592106

[15] Lu TH Chou YJ Liou CS. Impact of SARS on healthcare utilization by disease categories: implications for delivery of healthcare services. Health Policy. (2007) 83:375–81. 10.1016/j.healthpol.2007.03.001 17445942 PMC7132456

[16] Brooks SK Webster RK Smith LE Woodland L Wessely S Greenberg N. The psychological impact of quarantine and how to reduce it: rapid review of the evidence. Lancet. (2020) 395:912–20. 10.1016/S0140-6736(20)30460-8 32112714 PMC7158942

[17] Reger MA Stanley IH Joiner TE. Suicide mortality and coronavirus disease 2019-a perfect storm? JAMA Psychiatry. (2020) 77:1093–4. 10.1001/jamapsychiatry.2020.1060 32275300

[18] Ohlsson H Kendler KS. Applying causal inference methods in psychiatric epidemiology: a review. JAMA Psychiatry. (2019) 77:637–44. 10.1001/jamapsychiatry.2019.3758 31825494 PMC7286775

[19] Goncalves-Pinho M Mota P Ribeiro J Macedo S Freitas A. The impact of COVID-19 pandemic on psychiatric emergency department visits—a descriptive study. Psychiatr Q. (2020) 1–11. 10.1007/s11126-020-09837-z. [Epub ahead of print]. 32839923 PMC7445073

[20] Goldenberg MN Parwani V. Psychiatric emergency department volume during Covid-19 pandemic. Am J Emerg Med. (2020) 41:233–4. 10.1016/j.ajem.2020.05.088 32507569 PMC7263232

[21] Jeffery MM D'Onofrio G Paek H Platts-Mills TF Soares WE 3rd Hoppe JA. Trends in emergency department visits and hospital admissions in health care systems in 5 states in the first months of the COVID-19 pandemic in the US. JAMA Intern Med. (2020) 180:1328–33. 10.1001/jamainternmed.2020.3288 32744612 PMC7400214

[22] Pfefferbaum B North CS. Mental health and the Covid-19 pandemic. N Engl J Med. (2020) 383:510–2. 10.1056/NEJMp2008017 32283003

